# The Law Affecting Medical Practitioners.—I

**Published:** 1902-11-15

**Authors:** J. E. R. Stephens

**Affiliations:** Barrister-at-Law, Author of "Digest of Public Health Cases"


### The Institutional Workshop.

### THE LAW AFFECTING MEDICAL PRACTITIONERS.—I.

By J. E. R. Stephens, Barrister-at-Law, Author of "Digest of Public Health Cases."

### Historical and Introductory.

The practice of medicine has long obtained legal sanction and recognition in this country. Practitioners were at one time divided into three main branches, known respectively as physicians, surgeons or barber-chirurgeons, and apothecaries, whose functions and status were quite distinct, who were governed by different charters and statutes, and pursued their callings independently of, and sometimes in antagonism to, one another. Besides the above, there have always been, as there are still, persons who attended to human ailments without having any legally-recognised qualification for doing so. The union of all qualified persons, and their recognition as members of one profession, is modern. The statutes of the United Kingdom which have direct relation to medicine are: (1) those relating to the public health; (2) those relating to lunacy (and habitual drunkenness); (3) those relating to the status of the medical profession, to dentists and to pharmaceutical chemists; (4) those relating to restrictions on the "practice" of anatomy and physiology. There are, besides, several statutes in which medicine is concerned indirectly, such as the Poor Laws, the Prisons Acts, the Shipping Acts, the Registration of Births and Deaths Act, the Sale of Food and Drugs Act, the Sale of Poisons Act, the Factory and Workshops Act, the Rivers Pollution Prevention Act, the Contagious Diseases (Animals) Act, and the Public Health (Water) Act.

By a statute passed in 1511 (3 Hen. VIII. c. 11) no person was permitted to take upon him to exercise and occupy as a physician and surgeon until he had been examined and approved for the calling. The examination was in those days conducted under the sanction of, and the approval obtained from, the bishop of the diocese. Physicians obtained a charter of incorporation in the year 1518, and in 1522 the college got the right of examining and approving persons to practise physic, concurrently with graduates of the universities of Oxford and Cambridge. The bishop's licence was thus superseded (14 and 15 Hen. VIII. c. 5). The colleges were also empowered to exercise control and supervision over all dealers in drugs. By a subsequent Act of the same reign (32 Hen. VIII. c. 40), persons chosen and admitted by the President and Fellows of the College of Physicians were authorised to practise in physic, including surgery, anywhere within the realm. The charter and Act of 1522 were recognised and confirmed in 1553, and the privileges of the college enlarged (1 M. Sess. 2 c. 9). Their charter and privileges were again recognised by statute in 1858, 1859, 1860, and 1886.

Surgery was originally practised by barbers. In the City of London, the barbers obtained a charter of incorporation from King Edward IV. in the year 1461. Many other skilled surgeons were, however, in practise, and in the year 1540 they and the barbers were incorporated, and were given the exclusive right to practise surgery within the City of London and its suburbs (32 Hen. VIII. c. 42). Under the title of "The Mystery and Commonalty of Barbers and Surgeons of London," the physicians and barber-surgeons seem to have attempted to monopolise all treatment of disease to their own profit, for in the year 1452 (34 and 35 Hen. VIII. c. 9) an Act was passed declaring that it shall be lawful for any subject of the king "having knowledge and experience of the nature of herbs, roots, and waters, or the operation of the same, to practise, use and minister in and to any outward disorder, any herbs, ointments, bettes, pulters, and cornplaisters, according to their cunning, or drinks for the stone, strangury, or agues, without suit, vexation, trouble, penalty, or loss of their goods." The company obtained a further charter in the year 1629 (5 Car. I), giving it the right to examine and approve all persons who wished to practise surgery in London and Westminster, and empowering persons so approved to practise anywhere in England. In the year 1745, by 18 Geo. II. c. 15, the barbers and surgeons were separated into two companies. All the above liberties and privileges were transferred to the new Company of the Art and Science of Surgeons of London, who were empowered to make bye-laws, etc., for their own regulation, ordinance and government, and to examine candidates and authorise those who passed successfully to practise the art and science of surgery throughout His Majesty's dominions.

A statute passed in the year 1542 (33 Hen. VIII. c. 12) shows us very clearly the barbarous state of surgical practices at that period. The statute, after enacting that every person striking, and thereby shedding blood in the king's palace or house, shall have his hand stricken off, orders that there shall be present at the execution, among others, the chief surgeon for the time being, to sear the stump; the sergeant of the pantry to give bread to the person whose hand has been stricken off; the serjeant of the cellar to give to the same a draught of red wine after the searing; the serjeant of the ewry and the yeoman of the chandry with cloths and seared cloths respectively for the surgeon; the serjeant of the poultry with a cock ready for the surgeon to wrap about the bleeding stump; the yeoman of the scullery to prepare a fire and make ready searing irons; the chief ferror to bring with him searing irons and deliver them when hot to the surgeon; and the groom of the salcery with vinegar and cold water to give attendances upon the surgeon.

The medical profession seems to have been hardly so lucrative in the seventeenth century as at the present day, if we may judge by the records which we have of the fees usually paid at that time. Thus, in 1618, we find 2s. sent, with the urine, to an eminent physician; but, in another case, 6d. seems to have been considered sufficient. In 1700 the dues of graduates in physic amounted to 10s., but we hear that they expected 20s. The fee of a licensed physician was 6s. 8d., and of a surgeon 1s. per mile. The charge for bleeding was 1s., for setting a bone, broken or dislocated, 10 groats, and for amputation £5. In the time of Charles I. physicians used to visit their patients on horseback, sitting sideways.

The progress of medical knowledge in this country during the seventeenth century has been ably described by Lord Macaulay in his history of England (vol. i., pp. 310, 411). "Medicine," he says, "which, in France, was still in abject bondage, and afforded an inexhaustible subject of just ridicule to Molière, had in England become an experimental and progressive science, and every day made some new advance, in defiance of Hippocrates and Galen. The attention of speculative men had been, for the first time, directed to the important subject of sanitary police. To that period belong the chemical discoveries of Boyle and the first botanical researches of Sloane. One after another, phantoms which had haunted the world through ages of darkness fled before the light. Astrology and alchemy became jests. Soon there was scarcely a county in which some of the quorum did not smile contemptuously when an old woman was brought before them for riding on broomsticks or giving cattle the murrain."

The union of the different branches of the medical profession under one governing body was effected in the year 1858 by the Medical Act (21 & 22 Vict. c. 90). This Act was amended in 1859 (22 Vict. c. 21), 1860 (23 & 24 Vict. c. 7), 1876 (39 & 40 Vict. c. 40 and c. 41), and in 1886 (49 & 50 Vict. c. 48). These Acts are to be read together, and are known as the Medical Acts.

Only persons registered under the Medical Acts are entitled to call themselves legally qualified medical practitioners (21 & 22 Vict. c. 90, sect 34). They only are eligible for various appointments (sects. 33–36), and they only can give valid certificates in cases where certificates of a medical practitioner are required. Every person who shall be registered under the provisions of this Act shall be exempt, if he shall so desire, from serving on all juries and inquests whatsoever, and from serving all corporate, parochial, ward, hundred, and township offices, and from serving in the militia (sect. 35). If any person shall wilfully procure or attempt to procure himself to be registered under this Act, by making or producing or causing to be made or produced any false or fraudulent representation or declaration, either verbally or in writing, every such person so offending, and every person aiding and assisting him therein, shall be deemed guilty of a misdemeanour in England and Ireland, and in Scotland of a crime or offence punishable by fine or imprisonment, and shall, on conviction thereof, be sentenced to be imprisoned for any term not exceeding twelve months (sect. 39). The General Council shall cause to be published under their direction a book containing a list of medicines and compounds, and the manner of preparing them, together with the true weights and measures by which they are to be prepared and mixed, and containing such other matter and things relating thereto as the general council shall think fit, to be called "British Pharmacopœia," and the General Council shall cause to be altered, amended, and republished such pharmacopœia as often as they shall deem it necessary (sect. 54).

### Removal of Names from Register.

By the Medical Act (21 & 22 Vic. c. 90) the General Medical Council were established, one of their duties being to keep a register of medical practitioners. By section 29, "if any registered medical practitioner shall, after due inquiry, be judged by the General Council to have been guilty of infamous conduct in any professional respect, the General Council may, if they see fit, direct the registrar to erase the name of such medical practitioner from the register." It was held in *Albutt* v. *General Medical Council* (54 J. P. 36—C.A.) that if the Council, acting *bonâ fide*, and after due inquiry have adjudged a medical practitioner to have been guilty of infamous conduct in a professional respect, a court of law has no jurisdiction to review their decision. In *Partridge* v. *General Medical Council*, the plaintiff was a duly registered dentist. He had advertised his name in connection with a dental institution. The General Council considered such conduct amounted to "disgraceful conduct in a professional respect" within the meaning of the Act, and erased his name from the register. In an action for a mandamus to have the name reinstated, it was held that there were facts before the General Council on which they might come to the determination to erase the plaintiff's name from the register, and therefore the court would not interfere.

By section 26 of the Medical Act, 1858, any entry which shall be proved to the satisfaction of the general or branch council to have been fraudulently or incorrectly made, may be erased from the register by order in writing of the council. It was held in *Reg.* v. *General Medical Council* (9 W. R. 413) that the council might make such an order, where the registration had been made under the dispensing power given to them by section 46, as well as where it had been made by the registrar under section 15.

One test of whether a person has been guilty of "infamous conduct in any professional respect" is whether he has in the pursuit of his profession done something in regard to it which is reasonably regarded by his professional brethren of repute and competency as disgraceful and dishonourable. The General Medical Council, acting under the powers of the Medical Act, 1858, held an inquiry in which they adjudged a medical practitioner to be guilty of infamous conduct in a professional respect, and directed the registrar to erase his name from the register. The inquiry was impugned on the ground of the disqualification of one of the members on the ground of interest. It was held that in the absence of a pecuniary interest, the true question is whether the relation of the impugned member to the inquiry is such that he can be reasonably suspected of bias. (*Allinson* v. *General Medical Council*, 58 J. P. 542, C.A.). The General Medical Council, acting under the powers of the Medical Act, 1858, held an inquiry in which they adjudged a medical practitioner to be guilty of infamous conduct in a professional respect, and removed his name from the register of medical practitioners. The proceedings were instituted by the managing body of a company called the Medical Defence Union, whose object was to protect the characters of medical practitioners and to suppress and prosecute unauthorised practitioners. Two out of the 29 persons who held the inquiry were members of the Medical Defence Union, but not of the managing body of the union. It was held that the two members had not such an interest in the matter in question as to disqualify them from taking part in the inquiry; and the court refused to interfere with the decision of the council (*Leeson* v. *General Medical Council*, 38 W.R. 303—C.A.).

The publication of the minutes of the council, containing a report of their proceedings, comprising a statement that the name of a specified medical practitioner has been removed from the register on the ground that, in the opinion of the council, he has been guilty of infamous conduct in a professional respect, is, if the report be accurate, and published *bona fide* and without malice, privileged, and the medical practitioner cannot maintain an action for libel against the council in respect of the publication (*Albutt* v. *General Medical Council* supra).

(*To be continued.*)

